# Spatiotemporal Interpolation Methods for the Application of Estimating Population Exposure to Fine Particulate Matter in the Contiguous U.S. and a Real-Time Web Application

**DOI:** 10.3390/ijerph13080749

**Published:** 2016-07-25

**Authors:** Lixin Li, Xiaolu Zhou, Marc Kalo, Reinhard Piltner

**Affiliations:** 1 Department of Computer Sciences, Georgia Southern University, Statesboro, GA 30460, USA; marc.kalo@gmail.com; 2 Department of Geology and Geography, Georgia Southern University, Statesboro, GA 30460, USA; xzhou@georgiasouthern.edu; 3 Department of Mathematical Sciences, Georgia Southern University, Statesboro, GA 30460, USA; rpiltner@georgiasouthern.edu

**Keywords:** fine particulate matter (PM_2.5_), spatiotemporal interpolation, shape function, Inverse Distance Weighting (IDW), cross validation, population exposure, web application, visualization, real-time air pollution

## Abstract

Appropriate spatiotemporal interpolation is critical to the assessment of relationships between environmental exposures and health outcomes. A powerful assessment of human exposure to environmental agents would incorporate spatial and temporal dimensions simultaneously. This paper compares shape function (SF)-based and inverse distance weighting (IDW)-based spatiotemporal interpolation methods on a data set of PM_2.5_ data in the contiguous U.S. Particle pollution, also known as particulate matter (PM), is composed of microscopic solids or liquid droplets that are so small that they can get deep into the lungs and cause serious health problems. PM_2.5_ refers to particles with a mean aerodynamic diameter less than or equal to 2.5 micrometers. Based on the error statistics results of k-fold cross validation, the SF-based method performed better overall than the IDW-based method. The interpolation results generated by the SF-based method are combined with population data to estimate the population exposure to PM_2.5_ in the contiguous U.S. We investigated the seasonal variations, identified areas where annual and daily PM_2.5_ were above the standards, and calculated the population size in these areas. Finally, a web application is developed to interpolate and visualize in real time the spatiotemporal variation of ambient air pollution across the contiguous U.S. using air pollution data from the U.S. Environmental Protection Agency (EPA)’s AirNow program.

## 1. Introduction

Particulate matter (PM) is the generic term for a broad class of chemically and physically diverse substances that exist as discrete particles (liquid droplets or solids) over a wide range of sizes [1]. Some particulates occur naturally, originating from volcanoes, dust storms, forest and grassland fires, living vegetation, and sea spray. Some other particulates are made from human activities, such as the burning of fossil fuels in vehicles, power plants, and various industrial processes [2]. The United States Environmental Protection Agency (EPA) first established national ambient air quality standards for PM in 1971. The published evidence supports an association between PM and an increased risk of mortality. It has been shown that those with cardiovascular or respiratory conditions and the youth and elderly are the most susceptible to the adverse effects of PM. The pollutant class studied in this paper is specifically fine particulate matter, or PM_2.5_, which refers to particles with a mean aerodynamic diameter less than or equal to 2.5 micrometers. PM_2.5_ is considered one of the most unhealthy particulate air pollutants because it is more likely to be toxic and can be breathed more deeply into the lungs. PM_2.5_ has been associated with visibility reduction [3,4], acute stroke mortality [5], and daily mortality in many U.S. cities [6].

In order to find the association between air pollutants such as PM_2.5_ and health effects, researchers need to estimate pollutant concentrations in the continuous space-time domain. Since concentration values are typically measured only at discrete monitoring sites and at certain time instances, estimation of pollutant concentrations at unmeasured locations and times is needed. Implementing an appropriate interpolation method is critical to the assessment of relationships between air pollution exposure and health outcomes.

Spatial interpolation has been well developed and widely used in Geographic Information Systems (GIS). It is used to estimate values at unknown locations based upon values that are spatially sampled. Traditional spatial interpolation models have been extensively investigated over the years. Popular spatial interpolation methods are Inverse Distance Weighting (IDW) [7,8], shape functions [9,10], radial basis functions [11], spline [12], natural neighbor [13], trend surfaces [14], Kriging [15], model-data fusion (sometimes called *analysis*) [16,17], and optimal interplation [18]. IDW, shape functions, radial basis functions, splines, natural neighbor, and trend surfaces are deterministic methods. They provide no indication of the extent of possible errors. Their output is fully determined by the parameter values and the inputs. There are no strict assumptions about the variability or randomness of a feature. These methods are relatively simple to implement. On the other hand, Kriging, model-data fusion, and optimal interpolation are stochastic methods that possess some inherent randomness. The same set of parameter values and inputs will lead to an ensemble of different outputs. Stochastic methods provide probabilistic estimates. One of the advantages of stochastic methods is that they treat clusters more like single points and assign individual points within a cluster less weight than isolated data points, which helps to compensate for the effect of data clustering. In the field of atmospheric data analysis, model-data fusion and optimal interpolation methods are developed to include physics and chemistry of an air quality model in the interpolation mechanism and thus achieve better prediction and representation of air quality.

Nowadays, modern sensors are able to monitor different variables (such as particulate matter, sulfur dioxide, and ozone) at an increasing temporal resolution, resulting in rich spatiotemporal data sets. This calls for appropriate theories and methods to deal with these data sets to gain a better understanding of the observed spatiotemporal processes. Traditionally, many GIS researchers treat space and time separately [19]. They simply reduce spatiotemporal interpolation problems to spatial interpolation problems by assuming that time can be incorporated by conducting a sequence of snapshots of spatial interpolations. Since the spatiotemporal interpolation considers the additional time attribute, it can provide more accurate predictions than pure spatial interpolation. However, adding the temporal domain implies that variability in space and time must be modeled, which is more complicated than modeling purely spatial or purely temporal variability. A review of some air pollution exposure assessment methods utilized in epidemiological studies and the use of GIS for resolving problems with spatiotemporal attributes can be found in [20]. Other work on spatiotemporal interpolation are presented in the literature [9,21,22,23,24,25,26,27,28].

The main challenge presented by the spatiotemporal interpolation relates to the spatiotemporal dependence structure, i.e., the relative importance of time with reference to space. A powerful assessment of human exposure to air pollution would incorporate spatial and temporal dimensions. The temporal dimension of environmental exposure analysis is often ignored, underemphasized, or isolated from the spatial domain mainly due to the few efficient and effective tools to interpolate complex spatiotemporal datasets. The popular ArcGIS software (version 10.3, ESRI, Redlands, CA, USA) cannot handle spatiotemporal interpolation and is computationally inefficient with large datasets.

This paper has three goals. First, it investigates and compares two different spatiotemporal interpolation methods for an actual set of PM_2.5_ data measured by U.S. EPA monitoring sites in the contiguous United States: shape function (SF)-based vs. Inverse Distance Weighting (IDW)-based methods using the so-called *extension approach*. The extension approach has been proposed in [9] to integrate space and time simultaneously by extending spatiotemporal interpolation problems into higher dimensional spatial interpolation problems. SF and IDW are originally deterministic spatial interpolation methods. Since they can be extended to higher dimensions, they are both suitable for the extension approach. Furthermore, IDW is one of the most commonly used interpolation methods [7,23,29,30,31] for GIS applications. Although SF was initially from engineering, it has shown great interpolation performance in various GIS application data such as real estate data [9] and air pollution data [23,24,32]. Second, after obtaining the comparison results of the SF-based and IDW-based spatiotemporal interpolation methods, we apply the better method to estimate population exposure to PM_2.5_ in the contiguous United States using interpolated daily PM_2.5_ concentration values at the centroids of census block groups. Third, we aim to develop a web application to interpolate and visualize in real time the spatiotemporal variation of ambient air pollution (including but not limited to PM_2.5_) across the contiguous U.S. using air pollution data from the U.S. EPA’s AirNow program.

## 2. Methods

### 2.1. Shape Function-Based Spatiotemporal Interpolation Using the Extension Approach

Shape functions (SF) have been popular and utilized in engineering applications such as finite element algorithms [10,33]. Just like other traditional spatial interpolation methods used in GIS such as IDW [8] and Kriging [15], SF-based methods assume a stronger correlation among points that are closer than those farther apart. Therefore, SF-based methods can be spatial interpolation methods for GIS applications [9,25,34,35,36,37,38]. In addition, because the computational complexity of SF-based methods is linear, they can be efficient interpolation methods for large data sets.

#### 2.1.1. General Formula of the SF-Based 3D Spatial Interpolation Method

**Figure 1:**
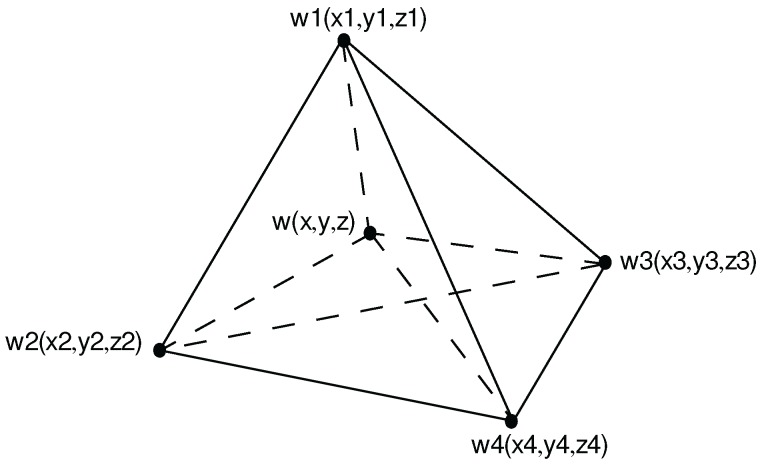
A tetrahedral element. Computing 3D shape functions by tetrahedral volume divisions. $w_{1}$, $w_{2}$, $w_{3}$ and $w_{4}$ are measured values, while the value *w* at the location (*x*, *y*, *z*) is unknown and needs to be interpolated.

In order to apply SF-based interpolation methods, a mesh that divides the total domain into a finite number of simple sub-domains or elements should be generated. For a 3D spatial problem, a mesh composed of tetrahedral elements should be generated if one wants to use shape functions for tetrahedra to interpolate unknown values in the 3D (x, y, z) coordinate system. Considering the tetrahedral element in Figure 1, the SF-based interpolation result *w* at an unknown point (*x*, *y*, *z*) located inside the tetrahedron can be obtained by using the measurement values $w_{1}$, $w_{2}$, $w_{3}$, and $w_{4}$ at the four known locations, which serve as the corner vertices of the tetrahedron as in [9]:

(1) $$\begin{array}{c} w\left(x,y,z\right)=N_{1}\left(x,y,z\right)w_{1}+N_{2}\left(x,y,z\right)w_{2}+N_{3}\left(x,y,z\right)w_{3}+N_{4}\left(x,y,z\right)w_{4} \end{array}$$

where $N_{1}$, $N_{2}$, $N_{3}$ and $N_{4}$ are the following linear shape functions:

(2) $$\begin{array}{c} N_{1}\left(x,y,z\right)=\frac{V_{1}}{V}\;,\;N_{2}\left(x,y,z\right)=\frac{V_{2}}{V}\;,\;N_{3}\left(x,y,z\right)=\frac{V_{3}}{V}\;,\;N_{4}\left(x,y,z\right)=\frac{V_{4}}{V} \end{array}$$

$V_{1}$, $V_{2}$, $V_{3}$ and $V_{4}$ are the volumes of the four sub-tetrahedra $ww_{2}w_{3}w_{4}$, $w_{1}ww_{3}w_{4}$, $w_{1}w_{2}ww_{4}$, and $w_{1}w_{2}w_{3}w$, respectively; and V is the volume of the bounding tetrahedron $w_{1}w_{2}w_{3}w_{4}$ as shown in Figure 1.

It can be seen from Figure 1 that $V_{1}$ is the volume of the sub-tetrahedron with four corner vertices as the unknown point (*x*, *y*, *z*) and three known points 2–4. Suppose the unknown point moves closer to the known point 1. $V_{1}$ is increasing, while $V_{2}$, $V_{3}$ and $V_{4}$ are decreasing, which lead to the increment of $N_{1}$ and decrement of $N_{2}$, $N_{3}$ and $N_{4}$. In an extreme case, when the unknown point moves to the exact location 1, the weight of $N_{1}$ becomes 1 and the other three weights $N_{2}$, $N_{3}$ and $N_{4}$ become 0. Similar observations can be made that any of the other three known points 2–4 will contribute a heavier weight in interpolating the value at the unknown point when the unknown point gets closer to this particular known point.

In finite element methods, shape functions of different orders (linear, quadratic, cubic, etc.) are used. In engineering, finite elements are used to approximate processes governed by differential equations such as deformations and stresses in a car. Whereas in engineering the nodal values at the corners of finite elements are all unknown and have to be computed from a large system of equations, in GIS applications, the nodal values at the element corner points come from measured data collections. The common point between finite element and GIS applications is that with nodal values the interpolation function can be evaluated for the complete domain. The size of the finite elements depends on the gradients and the changes in the function. For high gradients and oscillating functions, more elements of smaller size are needed. For the data interpolation, the situation is similar: if we expect high gradients and a lot of changes in a relatively small area, then we would ideally need a sufficiently high number of discrete data values to result automatically in a larger number of smaller elements.

#### 2.1.2. Extension Approach of the SF-Based Spatiotemporal Interpolation Method

Although spatial interpolation methods are well developed and widely adopted in various GIS applications [39,40,41,42] , the traditional spatial interpolation methods face many challenges when handling spatiotemporal data because of the addition of the time attribute of the data set. One of the major challenges is that traditional GIS researchers tend to treat space and time separately when interpolation needs to be conducted in the continuous space-time domain. The primary strategy identified from the literature is to reduce spatiotemporal interpolation problems to a sequence of snapshots of spatial interpolations [19]. However, integrating space and time simultaneously is anticipated to yield better interpolation results than treating them separately for certain typical GIS applications [43].

In order to integrate space and time simultaneously for a spatiotemporal interpolation, the *extension approach* has been proposed in [9] and reviewed in [37,38]. This approach treats time as another dimension in space, thereby extending the spatiotemporal interpolation problem into a higher-dimensional spatial interpolation problem. Applications using the extension approach can be found in [9,32,44,45]. To develop the extension approach for SF-based interpolation methods, we substitute the *z* variable in Equations (1) and (2) by ct, where *t* is the time variable and *c* is a factor of [spatial distance unit/time unit]. Equations (3) and (4) define our SF-based spatiotemporal interpolation method for 2D space and 1D time problems:

(3) $$\begin{array}{c} w\left(x,y,ct\right)=N_{1}\left(x,y,ct\right)w_{1}+N_{2}\left(x,y,ct\right)w_{2}+N_{3}\left(x,y,ct\right)w_{3}+N_{4}\left(x,y,ct\right)w_{4} \end{array}$$

where $N_{1}$, $N_{2}$, $N_{3}$ and $N_{4}$ are the following linear shape functions:

(4) $$\begin{array}{c} N_{1}\left(x,y,ct\right)=\frac{V_{1}}{V}\;,\;N_{2}\left(x,y,ct\right)=\frac{V_{2}}{V}\;,\;N_{3}\left(x,y,ct\right)=\frac{V_{3}}{V}\;,\;N_{4}\left(x,y,ct\right)=\frac{V_{4}}{V} \end{array}$$

Please note that there are some assumptions and resulting limitations for this approach. We assume that there are sufficient data measurements in space and time so that simple functions can be used to describe what is happening between two measurements. If there would be a relatively large time interval or data are scarcely sampled in space, and the type of data under consideration have the potential of strong oscillations between the points in space and time, we would not be able to use a simple linear function to interpolate from one space-time point to the next point. Therefore, before using this simple spatiotemporal approach, we have to make sure that the process that we analyze cannot show strong oscillations, and that we have sufficient measurements in space and time. We are only using the method to evaluate for events that already happened. We are not trying to predict the future with this method.

### 2.2. IDW-Based Spatiotemporal Interpolation Using the Extension Approach

Inverse Distance Weighting (IDW) is also known as Shepard’s method [7,8]. Similar to SF-based interpolation methods, IDW is based on Tobler’s First Law of Geography [46], which states: “Everything is related to everything else, but near things are more related than distant things”, page 236. IDW is generally considered a spatial interpolation method, but this paper applies IDW to spatiotemporal interpolation by using the *extension approach* and treating time as a third dimension [9,37].

#### 2.2.1. General Formula of the IDW-Based Spatial Interpolation Method

According to [47], the general formula of the IDW-based interpolation method in 2D space is:

(5) $$\begin{array}{c} w\left(x,y\right)=\sum_{i=1}^{N}\lambda_{i}w_{i},\;\;\;\lambda_{i}=\frac{{\left(\frac{1}{d_{i}}\right)}^{p}}{\sum_{k=1}^{N}{\left(\frac{1}{d_{k}}\right)}^{p}} \end{array}$$

where w(x,y) is the interpolated value at the unknown (or unsampled) location (x,y), *N* is the number of nearest known points surrounding (x,y), $w_{i}$ are the measurement values at the nearest known points of $\left(x_{i},y_{i}\right)$ (with 1≤i≤N), $\lambda_{i}$ are the weights assigned to $w_{i}$, $d_{i}$ are the Euclidean distances between each $\left(x_{i},y_{i}\right)$ and (x,y), and *p* is the exponent that influences the weighting of $w_{i}$ on *w*.

#### 2.2.2. Extension Approach of the IDW-Based Spatiotemporal Interpolation Method

The formula of the extension approach of IDW used in this paper is

(6) $$\begin{array}{c} w\left(x,y,ct\right)=\sum_{i=1}^{N}\lambda_{i}w_{i},\;\;\;\lambda_{i}=\frac{{\left(\frac{1}{d_{i}}\right)}^{p}}{\sum_{k=1}^{N}{\left(\frac{1}{d_{k}}\right)}^{p}} \end{array}$$

where

(7) $$\begin{array}{c} d_{i}=\sqrt{{\left(x_{i}-x\right)}^{2}+{\left(y_{i}-y\right)}^{2}+c^{2}{\left(t_{i}-t\right)}^{2}} \end{array}$$

and *c* is a factor defined as [spatial distance unit/time unit]. Compared with Equation (5), Equation (6) replaces w(x,y) with w(x,y,ct) and calculates the distance $d_{i}$ using the 3D Euclidean distance between $\left(x_{i},y_{i},ct_{i}\right)$ and (x,y,ct).

### 2.3. Cross Validation

The first goal of this paper is to compare whether SF-based or IDW-based spatiotemporal interpolation using the extension approach is more accurate in interpolating an actual set of data of daily fine particulate matter PM_2.5_ in the contiguous United States. K-fold cross validation [48] is used in this paper for this purpose.

#### 2.3.1. K-Fold Cross Validation

Classic validation divides the full data set into two data sets: a training data set and a validation data set. The validation data set is used for estimating the performance of the interpolation method based on the training data set. The interpolation method with the smallest error is selected as the best method. However, a potential flaw is that we may miss some characteristics in the full data set and make an inaccurate estimate of our model’s interpolation ability. Thus, k-fold cross validation is used to avoid this limitation. In this framework, the full data set is randomly split into k equal-sized data sets, with one group as the validation set and the remaining k-1 groups together forming the training set. This is repeated k times. In practice, 10-fold (k = 10) cross-validation is accepted as providing a highly accurate estimate of a model’s prediction errors. For large data sets, this approach may be computationally expensive.

Using 10-fold cross validation, the PM_2.5_ data set in our experiment is partitioned to ten nearly equally sized folds randomly. Ten iterations of training and validation are performed such that, within each iteration, a different fold of the data is held-out for validation while the remaining nine folds are used for learning. More specifically, within each iteration, the following two actions are taken:

1. The points in one fold (test data) of the PM_2.5_ data set are interpolated using the remaining nine folds (training data). Therefore, each point in the test data will have both the original PM_2.5_ concentration measurement and an interpolated PM_2.5_ concentration value.
2. Error statistics are calculated to compare the original and interpolated PM_2.5_ values in the test data.

#### 2.3.2. Error Statistics

The error statistics used in this paper are: MAE (Mean Absolute Error), MSE (Mean Squared Error), RMSE (Root Mean Squared Error) and MARE (Mean Absolute Relative Error). They are defined as follows:

(8) $$\begin{array}{c} MAE=\frac{\sum_{i=1}^{N}|I_{i}-O_{i}|}{N}\;\;\;\;\;\;\;\;\;\;MSE=\frac{\sum_{i=1}^{N}{\left(I_{i}-O_{i}\right)}^{2}}{N} \end{array}$$

$$\begin{array}{c} RMSE=\sqrt{\frac{\sum_{i=1}^{N}{\left(I_{i}-O_{i}\right)}^{2}}{N}}\;\;\;\;\;\;\;\;MARE=\frac{\sum_{i=1}^{N}\frac{|I_{i}-O_{i}|}{O_{i}}}{N} \end{array}$$

where *N* is the number of observations, $I_{i}$ is the interpolated value, and $O_{i}$ is the original measurement value. For each iteration of 10-fold cross validation, we have the assumption that a different set of training data has true measurements. From the mathematical point of view, it is reasonable to calculate averages of 10 sets of error statistics. We use $\bar{MAE}$, $\bar{MSE}$, $\bar{RMSE}$ and $\bar{MARE}$ to denote the average error statistics results in this paper.

In addition, we use an $R^{2}$ error statistic, which is also known as *the coefficient of determination*. The regular $R^{2}$ error statistic measures how close the data are to the fitted regression line, whereas the $R^{2}$ in [49] measures how close the data are to the 1–1 line. In this paper, we use the $R^{2}$ error statistic defined in [49]:

(9) $$\begin{array}{c} R_{CV}^{2}=max\left(0,1-\frac{RMSE^{2}}{MSE_{obs}}\right)\;\;\;\;\;\;\;\;\;\;MSE_{obs}=\frac{\sum_{i=1}^{N}{\left(O_{i}-\bar{O}\right)}^{2}}{N} \end{array}$$

where $\bar{O}$ is the mean of the original values. As for the other error statistics in Equation (8), the average of the ten $R_{CV}^{2}$ results needs to be calculated. We use $\bar{R_{CV}^{2}}$ to denote the average $R_{CV}^{2}$ result in this paper.

### 2.4. Linking PM_2.5_ to Census Population

We collected population data at the census block group level. To map PM_2.5_ and population spatial distribution, we created choropleth maps based on the interpolated values as well as population data. Because PM_2.5_ may exhibit different spatial patterns in different seasons, we also investigated the seasonal variations. In order to associate the PM_2.5_ values with the national standard, the revised U.S. EPA National Ambient Air Quality Standards for PM_2.5_ in 2006 were adopted in this paper. We conducted spatial queries to identify areas where annual and daily PM_2.5_ are above the standard and calculated the population size in these areas.

## 3. Experimental Data

The data used in this study are daily PM_2.5_ concentrations measured in 2009 by U.S. EPA monitoring sites in the contiguous United States.

### 3.1. PM_2.5_ Data Set with Measurements

**Figure 2:**
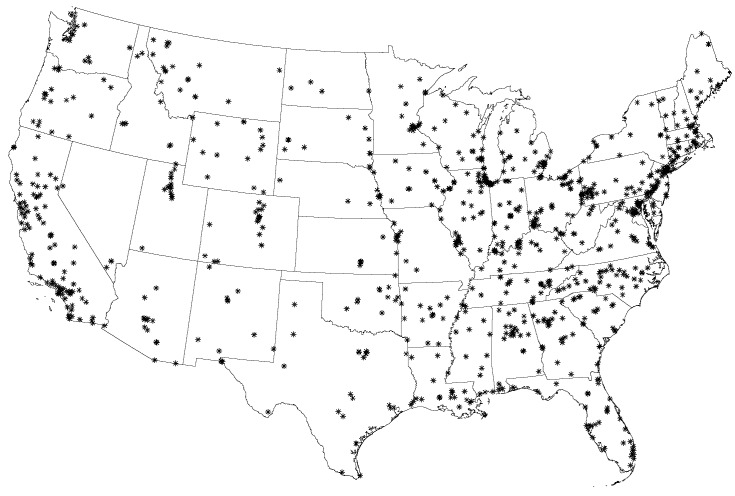
U.S. Environmental Protection Agency (EPA) monitoring sites. These monitoring sites have PM_2.5_ (fine particulate matter) measurements across the contiguous United States in 2009.

The data coverage contains locations of the monitoring sites, the daily concentration measurements of PM_2.5_, and the days of the measurements. We obtained a number of data sets from the U.S. EPA website [50] and reorganized them into a data set with the schema (id, *x*, *y*, [time], *w*), where *x* and *y* are the longitude and latitude coordinates of the monitoring sites, [time] is (year, month, day) when a PM_2.5_ measurement is taken, and *w* is the measured PM_2.5_ value. The reorganized data set has some entries with zero PM_2.5_ values, which means no measurements were available at a particular site and on a particular day. After all the zero entries are deleted, there are 146,125 daily measurements at 955 monitoring sites. The monitoring sites are illustrated as stars (*) in Figure 2.

### 3.2. Census Block Group Data Set to Interpolate

In our experiment, we want to interpolate daily PM_2.5_ concentration values in 2009 at the centroids of all the 207,630 census block groups in the contiguous United States. Census block groups are statistical divisions of census tracts and are generally defined to contain between 600 and 3000 people. They are the smallest geographical unit for which the United States Census Bureau publishes sample data. Our experimental data set with locations to compute interpolation has the format of (id,x,y) with id as the identification number of a census block group and (x,y) as the longitude and latitude coordinates of the centroid of a census block group. Since PM_2.5_ concentration values at the centroid of each census block group and on each day in 2009 are not measured, there are 207,630 × 365 = 75,784,950 PM_2.5_ values to be interpolated.

The motivation of interpolating at the small geographic level of the census block group is that we aim to link the interpolation results with the census block group population data in the same year for the second goal of this paper. As discussed in the Results section of the paper, we analyze population exposure to PM_2.5_ and estimate the U.S. population with unhealthy PM_2.5_ exposure. In future work, such estimates are important and we plan to link them to a variety of health outcomes to evaluate PM_2.5_’s adverse impact on human health.

## 4. Results

### 4.1. Cross Validation Results of the SF-Based Method

#### 4.1.1. Choice of Time Scale

In order to decide on an appropriate time scale for the SF-based method using the extension approach, we tested four time scales as shown in Table 1. The factor *c* in the table is from Equations (3) and (4).

A challenge of using the extension approach for spatiotemporal interpolation is the correlation between space and time, and which choice of the factor *c* is optimal for a particular data set. This is an open question and a research topic in GIS that has been rarely studied. In this paper, authors tested only four possible time scales in Table 1. More research is needed to address this challenge in the future.

#### 4.1.2. Cross Validation and Error Statistics

Ten-fold cross validation was implemented to test the four time scales in Table 1. Since there are ten iterations in 10-fold cross validation and a different fold of the data is held-out for validation during each iteration, the average of ten error statistics has been calculated for each error statistic in Equation (8).

Table 2 shows the results for the average error statistics ($\bar{MAE}$, $\bar{MSE}$, $\bar{RMSE}$, $\bar{MARE}$, and $\bar{R_{CV}^{2}}$) using the SF-based extension method for the PM_2.5_ data set. All of these five measures of error statistics are based on interpolated and original values, $I_{i}$ and $O_{i}$ in Equations (8) and (9), but they have different sensitivity to error patterns. The ideal situation is that $\bar{MAE}$, $\bar{MSE}$, $\bar{RMSE}$, and $\bar{MARE}$ are lowest, while $\bar{R_{CV}^{2}}$ is the highest for the same time scale choice. If not, we need to make a choice according to the characteristics of the five error measures. MSE, RMSE, and $R_{CV}^{2}$ are sensitive to individual outliers. MAE is less sensitive to outliers but could not reflect the relative prediction errors. MARE is less sensitive to outliers and also incorporates the predictive mean to measure the error from a model prediction. The same size of an error is not acceptable for a small predicted mean but could be acceptable for a large predicted mean. MARE is a better choice to evaluate overall model performance. However, if outliers are major concerns, RMSE or $R_{CV}^{2}$ would be better choices.

**Figure 3:**
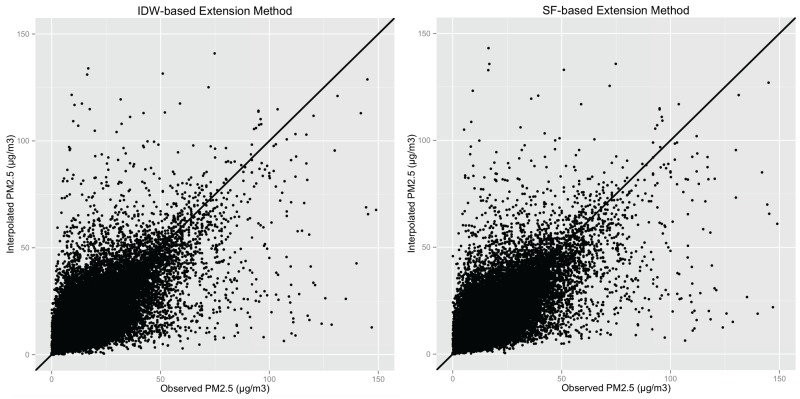
Scattered plots. Comparing observed daily PM_2.5_ values with interpolated daily PM_2.5_ values across monitoring sites across the contiguous United States in 2009.

We produced a scattered plot to compare observed daily PM_2.5_ values with interpolated daily PM_2.5_ values across monitoring sites. Please see Figure 3. Descriptive statistics show that the original PM_2.5_ values contain 16 outliers with PM_2.5_ values above $250\;\mu g/m^{3}$, which were much higher than the normal range. According to the National Ambient Air Quality Standards (NAAQS) established by the U.S. EPA under authority of the Clean Air Act, the 24 h standard for PM_2.5_ is met if the three-year average of the annual 98th percentile of values at designated monitoring sites in an area is less than or equal to $35\;\mu g/m^{3}$ [51]. The PM_2.5_ values above $250\;\mu g/m^{3}$ might be wrongly recorded or some short and extreme conditions happened. These conditions are not usual, so we removed these 16 outliers with PM_2.5_ values greater than $250\;\mu g/m^{3}$ from the original 146,124 values. The new error statistics result after removing the outliers are recorded in Table 3.

Compared with Table 2, Table 3 shows better error statistics for all measures. *Scale C* outperformed the other three scales on all error statistics, except for *Scale A* on $\bar{MAE}$. However, *Scale A* performed significantly poorly on $\bar{MARE}$, $\bar{RMSE}$, $\bar{R_{CV}^{2}}$, and $\bar{MSE}$. Thus, *Scale C* is selected as the best time scale for daily PM_2.5_ interpolation using the SF-based extension method.

### 4.2. Cross Validation Results of the IDW-Based Method

#### 4.2.1. Choice of Time Scale, Number of Neighbors, and Exponents

In order to choose an appropriate time scale for the IDW-based method using the extension approach and compare it with the SF-based method, the same four times scales in Table 1 were tested for the IDW-based method.

We evaluated 45 IDW methods with five choices for the number of nearest neighbors *N* (3, 4, 5, 6 and 7) and nine choices for the exponent *p* (1.0, 1.5, 2.0, 2.5, 3.0, 3.5, 4.0, 4.5 and 5.0).

#### 4.2.2. Cross Validation and Error Statistics

Similar to evaluating the SF-based method, 10-fold cross validation was implemented to test the time scales, as well as the choices for the number of nearest neighbors *N* and the exponent *p*. The optimal average error statistics among the forty-five combinations of *N* and *p* are summarized in Table 4 for each chosen time scale, along with the values of *N* and *p* when the optimal averages were obtained. Based on Table 4, we choose *Scale B* as the best of the four time scales for the IDW-based method since it provides the lowest $\bar{MARE}$, $\bar{MSE}$, and $\bar{RMSE}$, as well as the second highest $\bar{R_{CV}^{2}}$.

The decision of what values of *N* and *p* to use in order to achieve the best IDW interpolations possibly depends on the error statistic deemed most important to optimize. It should be noted that [31] also discussed the character of the exponent and suggested that the exponent should be deduced from the form of pollution encountered. For air pollution, [31] suggests that elementary reasoning shows that the exponent should be 2 or 3, but more sophisticated considerations could show that the exponent may vary between 1 and 3. For our study, the best exponent could depend on the specific outcome or measure we wanted to model. Hence, we experiment with different exponents *p* (1.0, 1.5, 2.0, 2.5, 3.0, 3.5, 4.0, 4.5 and 5.0) in order to select the one with the best performance via error analysis. If it were only possible to run an interpolation for one choice of the number of nearest neighbors *N* and the exponent *p* (because of time constraints, lack of computational resources, etc.), then the configurations of (N=7, p=1.0) and (N=3, p=5.0) seem better than the other configurations that were tested. The configuration of (N=7, p=1.0) yields the second highest $\bar{R_{CV}^{2}}$ among all time scales with a very close result to the highest $\bar{R_{CV}^{2}}$, whereas the configuration of (N=3, p=5.0) yields the least $\bar{MARE}$ among all time scales. In order to further investigate the difference between configurations of (N=7, p=1.0) and (N=3, p=5.0) under *Scale B*, we conducted a further experiment to compare just these two configurations.

The comparison results are shown in the first two columns of Table 5. We consider the configuration of (N=3, p=5.0) better than (N=7, p=1.0) under *Scale B* because (N=3, p=5.0) yields a smaller $\bar{MARE}$. Similar to the SF-based interpolation method, we removed the same outliers, recomputed the error statistics for the configuration of (N=3, p=5.0) under *Scale B*, and recorded them in the third column of Table 5. All of the error statistics improved after removing outliers.

### 4.3. Comparison of SF-Based and IDW-Based Extension Methods

The first goal of this paper is to compare the performance of the SF-based and IDW-based spatiotemporal interpolation methods in order to find the most suitable method for the PM_2.5_ data. It is evident from the error statistics, as shown in Table 2 and Table 3 for the SF-based method and Table 4 and Table 5 for the IDW-based method, that *Scale C* using the SF-based method is the best interpolation method among all the methods that we have tested for the PM_2.5_ data set. Both the SF-based and IDW-based methods see improvements in the accuracy of all error statistics, except $\bar{MAE}$, when choosing a different time scale than *Scale A*, with significant improvement of $\bar{MARE}$. The SF-based method outperforms the IDW-based method even in the IDW-based method’s best scenarios, i.e., the combinations of the number of nearest neighbors and exponents that minimize the relevant error statistics. Therefore, we choose the SF-based extension method using *Scale C* to interpolate the PM_2.5_ data set for population exposure analysis.

In addition to accuracy comparison based on cross validation, the SF-based spatiotemporal interpolation method using the extension approach is computationally efficient because the algorithm is linear according to Equations (3) and (4). On the other hand, the IDW-based method is non-linear according to Equation (6). Therefore, the IDW-based spatiotemporal interpolation method is not as computationally efficient as the SF-based method.

### 4.4. Population Exposure Analysis

The second goal of this paper is to evaluate the population exposure to fine particulate matter PM_2.5_ in the contiguous United States. Annually updated population data are only available from the five-year American Community Survey at the census block group level. Therefore, we used census block groups in our analysis. The SF-based spatiotemporal interpolation using *Scale C* and the extension approach was implemented to compute a total of 75,784,950(207,630×365) PM_2.5_ values at the centroids of 207,630 census block groups in the contiguous U.S. on each day in 2009. The interpolated census block group-level PM_2.5_ was then linked to 2009 census block group population data.

**Figure 4:**
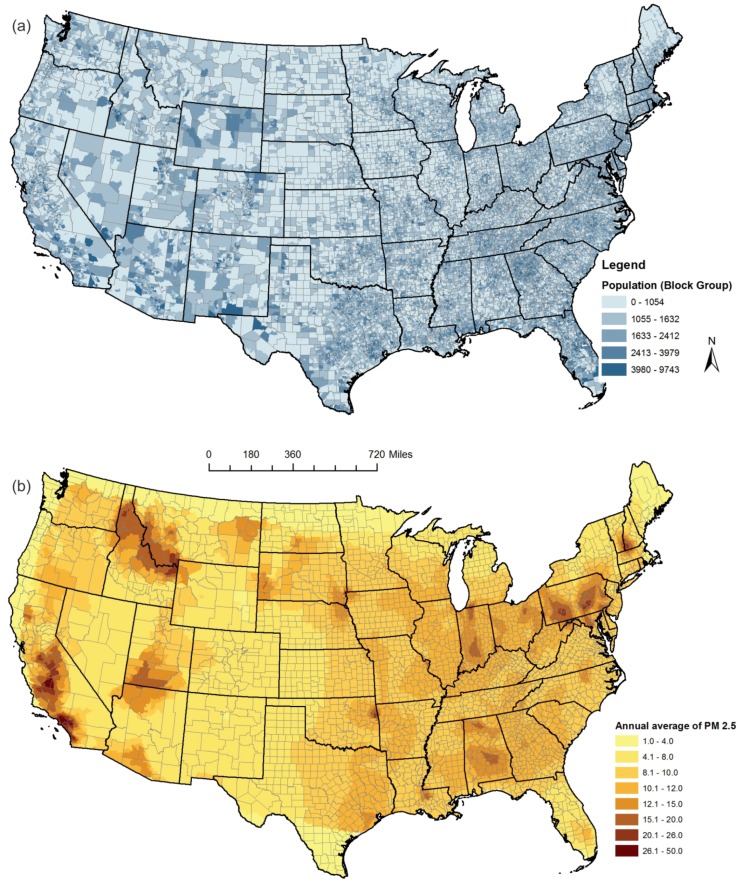
Spatial relationship between the PM_2.5_ concentration and the population distribution across the contiguous United States in 2009. (**a**) population distribution; (**b**) annual average PM_2.5_. We used natural breaks to define the color ramps. A lighter color represents a smaller value, while a darker color represents a higher value.

To analyze the spatial relationship between the PM_2.5_ concentration and the population distribution at the census block group level, we first plot the population distribution in Figure 4a. Second, we plot the annual PM_2.5_ average values in Figure 4b. Several hotspots of high PM_2.5_ values, such as central south California, the Idaho–Montana border, and some regions in Pennsylvania, are distinctively shown in Figure 4b.

**Figure 5:**
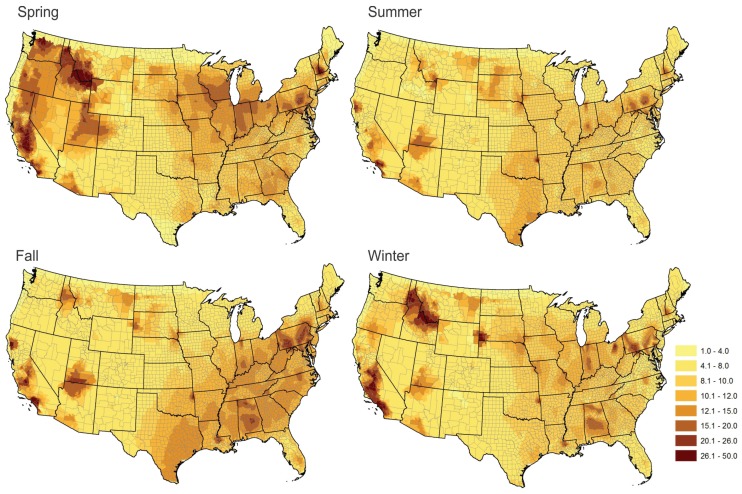
Seasonal differences. The average PM_2.5_ in different seasons across the contiguous United States in 2009. In order to make the color scheme consistent in four seasons, we manually defined the classification scheme. The legend shows the class ranges.

To investigate whether this pattern varies in different seasons, we break the annual average into seasonal averages. Because we used 2009 data, January, February, and December are combined as the winter season. Spring starts from March and ends in May. Summer starts from June and ends in August. The rest of the time is the fall season. Figure 5 shows the seasonal differences. We find that, in spring, the average PM_2.5_ values were high in the west and mountainous areas. The values substantially decreased in summer. In fall, the values increased in the southeast region. Some areas such as central south California had high PM_2.5_ values almost all year around.

**Figure 6:**
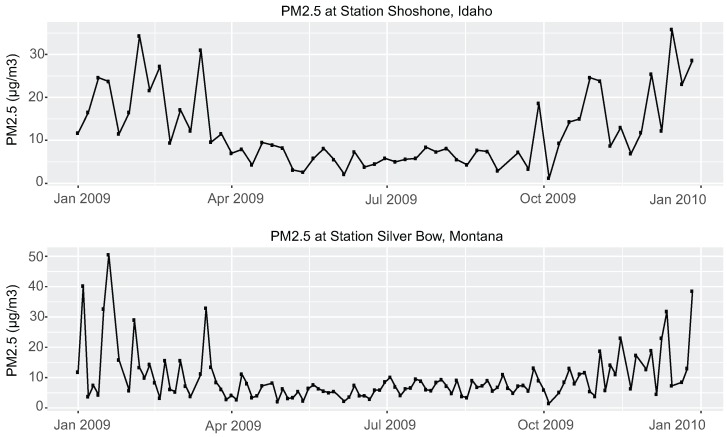
Verification of a spatial pattern in a region near the Idaho–Montana border in 2009. Plots of PM_2.5_ measurements at two monitoring stations in Idaho and Montana using PM_2.5_ daily data from AirNow in 2009.

In addition, we observe from Figure 5 that a region near the Idaho–Montana border shows higher PM_2.5_ values during spring and winter than during summer and fall of the year 2009. To verify this pattern, we used the PM_2.5_ Federal Reference Method (FRM)/Federal Equivalent Method (FEM) Mass (88101) daily data (arithmetic mean value) from AirNow to plot the PM_2.5_ values in 2009 at two monitoring stations in Idaho and Montana, as shown in Figure 6. The trends at these two stations are consistent with what we observed in Figure 5. The reason for this pattern remains unclear, despite efforts elucidate its cause. More investigation on the cause for high PM_2.5_ values in this region in 2009 is needed in the future.

Finally, in order to associate the PM_2.5_ values with the national standard, the revised U.S. EPA National Ambient Air Quality Standards for PM_2.5_ in 2006 [51] were adopted in this paper:

- 35 micrograms per cubic meter ($35\;\mu g/m^{3}$) for 24 h:
  We identify block groups that have PM_2.5_ values greater than $35\;\mu g/m^{3}$ for at least one day.
- 15 micrograms per cubic meter ($15\;\mu g/m^{3}$) for the annual mean:
  We identify block groups that have annual PM_2.5_ values greater than $15\;\mu g/m^{3}$.

**Figure 7:**
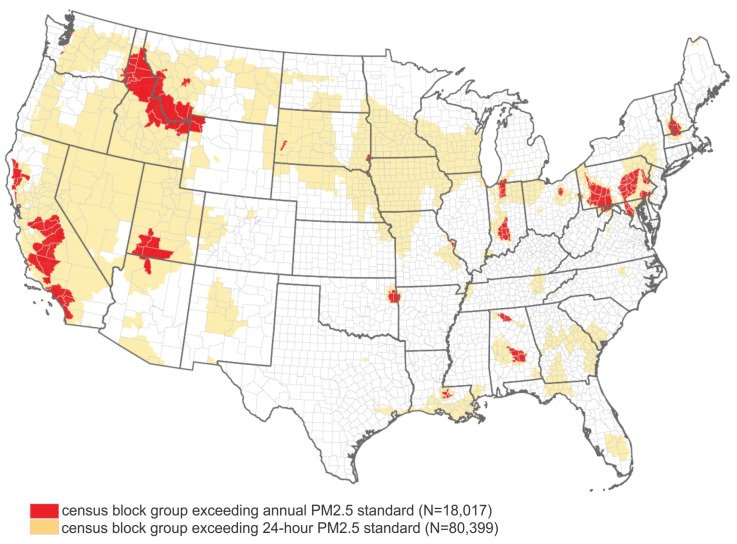
Geographic distribution of census block groups in the contiguous United States that exceeded the PM_2.5_ air quality standards in 2009.

Figure 7 shows the geographic distribution of such census block groups with the annual and/or 24 h exceeding the U.S. EPA National Ambient Air Quality PM_2.5_ standards. The results suggest:

- there is a population of 27,809,017 (27.8 million) residing in 18,017 census block groups in the contiguous United States with an annual PM_2.5_ exceeding the national standard of $15\;\mu g/m^{3}$;
- more than one-third of the U.S. population (115,310,354) residing in 80,399 census block groups where PM_2.5_ exceeded $35\;\mu g/m^{3}$ for at least one day in 2009.

### 4.5. Web Application

The third goal of this paper is to develop a web application to interpolate and visualize in real time the spatiotemporal variation of ambient air pollution (including but not limited to PM_2.5_) across the contiguous U.S. The web application is based on the MEAN framework [52]. This framework relies on the MongoDB database [53] to store the application’s data, Express framework [54] to facilitate HTTP routing, AngularJS [55] to construct an MVC (Model View Controller) architecture to simplify building of responsive web pages, and NodeJS [56] to support the application. The use of MongoDB, Node.js, Express, and AngularJS provides a unified development approach. Each of the technologies is based on JavaScript which allows for more code reuse and less context switching for developers as they move between server side and client side application development.

In addition, a REST (REpresentational State Transfer) [57] Application Program Interface (API) is utilized to handle requests from clients, including user sign up and authentication, requests for interpolated pollution data, and requests for triangulations of measurement sites. A REST call is used to initiate the downloading of pollution data from the *AirNow* [58] File Transfer Protocol (FTP) server and initiate the triangulation and interpolation of the data using the SF-based method. AirNow is a U.S. EPA program that provides real-time observed air quality information across the U.S., Canada, and Mexico. It receives real-time air quality observations from over 2,000 monitoring stations and collects forecasts for more than 300 cities. The AirNow program includes a web services API for accessing current and historical pollution data [59]. However, queries to this service are generally rate limited to 500 per hour. Therefore, the web application presented in this paper uses an alternative FTP server method to access the AirNow data.

This web application uses an SF-based interpolation to compute and update any hour/parameter combination when data has not been updated. Using this method, the system can always include the data for the latest downloaded hour and may include data for previous hours if a time-based interpolation has been calculated. Triangulations are stored in the MongoDB database in a “triangles” collection. When a query is received, the web application can use a geospatial query supported by MongoDB to locate the containing triangle in the triangulation and interpolate the PM_2.5_ concentration.

**Figure 8:**
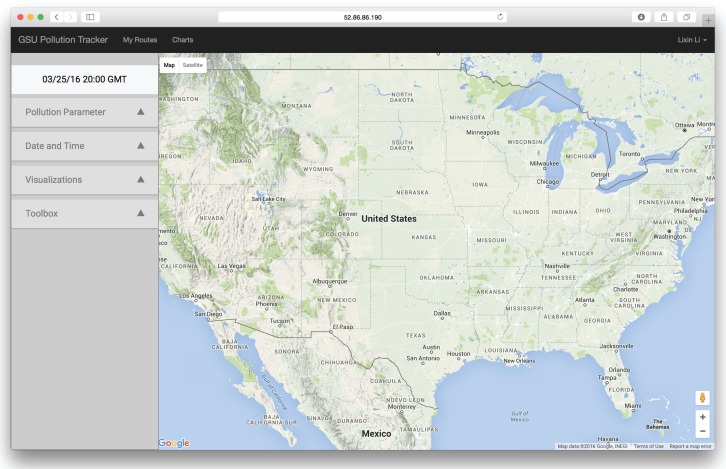
Web application. Map overview screen after logging in.

In order to use the web application, the user needs to sign up by filling out a simple form or log in if they already have an account at the website [60]. After successful log in, the user will see the screen in Figure 8. The screen includes an options menu on the left and an embedded Google Maps application on the right. The Google Maps application is the main panel used for visualization of pollution data, developed using the Google Maps API. When the user changes visualization options in the options menu, such as selecting the pollution parameter type, date, time, or visualization rendering parameters, the data in the Google Maps application will be updated automatically and responsively rendered.

**Figure 9:**
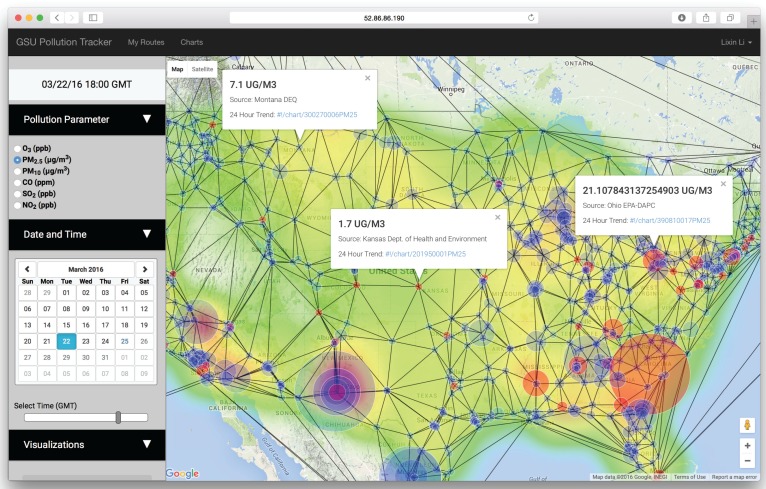
Web application. Rendering of PM_2.5_ concentrations across the contiguous U.S. on 22 March 2016 at 18:00 GMT, including intensities at known measurement sites and the resultant triangulations used in the shape function (SF)-based interpolation method.

Visualization of the pollution data is rendered on the client side by embedding a Google Maps application within the AngularJS application. Figure 9 shows the interpolated PM_2.5_ concentrations across the contiguous U.S. on 22 March, 2016 at 18:00 GMT. This web application allows a user to visualize six air pollution parameters: O_3_ (ppb), PM_2.5_ ($\mu g/m^{3}$), PM_10_ ($\mu g/m^{3}$), CO (ppm), SO_2_ (ppb), and NO_2_ (ppb).

## 5. Discussion

Due to the technological advances and the societal need for analysis of physical phenomena that continuously change in space and time, such as weather and air quality variables, etc., the collection and processing of spatiotemporal data becomes more and more important. There are significant spatial and temporal dependencies among these data, which are usually ignored or underemphasized by a purely spatial interpolation approach. Investigating the additional temporal information has the potential to improve the interpolation result. Therefore, developing appropriate spatiotemporal interpolations is critical to estimate missing values at points from neighboring observations by looking deep into the spatial and temporal correlations.

This study compares performance of the SF and IDW based spatiotemporal interpolation methods in order to find an interpolation method suitable for an actual set of daily PM_2.5_ values in the contiguous U.S. This paper also explored population exposure to PM_2.5_ in the contiguous U.S. by linking interpolated PM_2.5_ at the centroids of census block groups to census population. Finally, we implemented a web application to interpolate and visualize in real time the spatiotemporal variation of ambient air pollution (including but not limited to PM_2.5_) across the contiguous U.S. using air pollution data from the U.S. EPA’s AirNow program. There are some limitations and future work with our study:

- This study is limited to investigating only four choices for time scales, five choices for the number of nearest neighbors, and nine choices for the exponents. In future work, we plan to apply machine learning methods to efficiently learn the best possible configurations in the model, using a lightning-fast cluster computing framework *Apache Spark* [61].
- The SF-based and IDW-based methods are deterministic methods. In this paper, we did not compare our methods with geostatistical interpolation methods such as Kriging, neural networks, and land use regression. In future work, we plan to develop multidimensional and stochastic spatiotemporal interpolation methods suitable for ambient air pollution data (NO_2_, O_3_, PM_2.5_, and PM_10_) by incorporating factors associated with the environmental exposure of interest, and then make comparisons with other commonly-applied geostatistical interpolation methods.
- Finally, there is a limitation in the currently implemented SF-based algorithm with respect to missing data close to some boundaries of the contiguous United States. For example, along the west coast in Oregon and Washington, there are monitoring stations relatively far away from the coastal border. Because of missing data, an unrealistic stripe next to the coast is visible in our map presentations of the interpolated results. In order to avoid this type of problem, we will need additional measurements along the coast, or use meshless interpolation methods such as IDW with a limited number of neighboring measurements in future work.

Additionally, in future work, we plan to link interpolated air pollution concentration values to a variety of health outcomes to evaluate air pollution’s adverse impact on human health, as well as link the interpolated pollution values with individual GPS trajectory to better estimate personal-based air pollution exposure.

## 6. Conclusions

In conclusion, this study has made three contributions to the ambient air pollution and spatiotemporal interpolation research community.

First, using an actual set of daily PM_2.5_ values measured by U.S. EPA monitoring sites in the contiguous United States, the performance of the SF and IDW based spatiotemporal interpolation methods is compared in order to find an interpolation method suitable for the PM_2.5_ data. The SF-based interpolation method performed better overall than the IDW-based method for the daily PM_2.5_ data.

Second, more than 75 million PM_2.5_ spatiotemporal interpolation results are calculated using the SF-based spatiotemporal method in the contiguous U.S. at the fine geographic level of census block groups. The interpolation results are linked to 2009 census block group population data so that the population with unhealthy PM_2.5_ exposure in the contiguous U.S is estimated. To map PM_2.5_ and population spatial distribution, we generated choropleth maps based on the interpolated values as well as the population data. Because PM_2.5_ may exhibit different spatial patterns in different seasons, we also investigated the seasonal variations. We conducted spatial queries to identify areas where annual and daily PM_2.5_ are above the standard and calculated the population size in these areas.

Third, this study implemented a web application to interpolate and visualize in real time the spatiotemporal variation of ambient air pollution (including but not limited to PM_2.5_) across the contiguous U.S. using air pollution data from the U.S. EPA’s AirNow program.

## Figures and Tables

**Table 1:** Four times scales tested for the PM_2.5_ (fine particulate matter) data set.

Time	Scale A	Scale B	Scale C	Scale D
	(*c* = 1)	(*c* = 1/10)	(*c* = 1/5)	(*c* = 1/15)
01/01/2009	1	0.1	0.2	0.067
01/02/2009	2	0.2	0.4	0.133
01/03/2009	3	0.3	0.6	0.2
01/04/2009	4	0.4	0.8	0.267
…	…	…	…	…
12/31/2009	365	36.5	73	24.333

**Table 2:** Error statistics for the PM_2.5_ data set using the shape function-based extension method and 10-fold cross validation before removing outliers.

Error	Scale A	Scale B	Scale C	Scale D
Statistics	(c=1)	(c=1/10)	(c=1/5)	(c=1/15)
$\bar{MAE}$	3.1512	3.5576	3.2463	3.7307
$\bar{MSE}$	85.8621	78.5322	78.4890	77.1072
$\bar{RMSE}$	8.8832	8.6045	8.6067	8.5023
$\bar{MARE}$	3.2162	0.4158	**0.3745**	0.4365
$\bar{R_{CV}^{2}}$	0.3079	0.3226	0.3138	0.3382

**Table 3:** Error statistics for the PM_2.5_ data set using the shape function-based extension method and 10-fold cross validation after removing outliers.

Error	Scale A	Scale B	Scale C	Scale D
Statistics	(c=1)	(c=1/10)	(c=1/5)	(c=1/15)
$\bar{MAE}$	3.0941	3.4976	3.1812	3.6751
$\bar{MSE}$	42.2910	37.7745	**35.6601**	39.2077
$\bar{RMSE}$	6.5032	6.1461	**5.9716**	6.2616
$\bar{MARE}$	3.2135	0.4128	**0.3708**	0.4349
$\bar{R_{CV}^{2}}$	0.4817	0.5371	**0.5630**	0.5195

**Table 4:** Error statistics for the PM_2.5_ data set using the IDW-based extension method and 10-fold cross validation. $\bar{MAE}$ (Mean Absolute Error), $\bar{MSE}$ (Mean Squared Error), $\bar{RMSE}$ (Root Mean Squared Error), $\bar{MARE}$ (Mean Absolute Relative Error) and $\bar{R_{CV}^{2}}$ are the optimal averages of the error statistics.

Error	Scale A	Scale B	Scale C	Scale D
Statistics	(c=1)	(c=1/10)	(c=1/5)	(c=1/15)
	3.1586	3.2856	3.1070	3.4207
$\bar{MAE}$	(*N* = 4, *p* = 1.0)	(*N* = 3, *p* = 2.0)	(*N* = 3, *p* = 2.0)	(*N* = 5, *p* = 2.5)
	75.3792	67.8379	68.0293	68.2309
$\bar{MSE}$	(*N* = 7, *p* = 1)	(*N* = 7, *p* = 1.5)	(*N* = 6, *p* = 1.0)	(*N* = 7, *p* = 1.5)
	8.3258	7.8888	7.8967	7.9143
$\bar{RMSE}$	(*N* = 7, *p* = 1.0)	(*N* = 7, *p* = 1.5)	(*N* = 7, *p* = 1.0)	(*N* = 7, *p* = 1.5)
	2.7005	**0.3803**	0.9717	0.3963
$\bar{MARE}$	(*N* = 7, *p* = 1.0)	**(*N* = 3, *p* = 5.0)**	(*N* = 3, *p* = 5.0)	(*N* = 3, *p* = 2.5)
	0.3789	**0.4413**	0.4416	0.4374
$\bar{R_{CV}^{2}}$	(*N* = 7, *p* = 1.0)	**(*N* = 4, *p* = 1.0)**	(*N* = 7, *p* = 1.0)	(*N* = 7, *p* = 1.0)

**Table 5:** Error statistics comparison of two configurations under *Scale B* using the IDW-based extension method and 10-fold cross validation.

Error Statistics	N=7,p=1.0	N=3,p=5.0	N=3,p=5.0
	Scale B (c=1/10)	Scale B (c=1/10)	Scale B (c=1/10)
	before Removing Outliers	before Removing Outliers	after Removing Outliers
$\bar{MAE}$	3.4519	3.3378	**3.2765**
$\bar{MSE}$	68.0348	79.5497	**37.5608**
$\bar{RMSE}$	7.8909	8.6320	**6.1287**
$\bar{MARE}$	1.2594	**0.3803**	**0.3773**
$\bar{R_{CV}^{2}}$	0.4413	0.3359	**0.5399**
